# Are Normal Decision-Makers Sensitive to Changes in Value Contrast under Uncertainty? Evidence from the Iowa Gambling Task

**DOI:** 10.1371/journal.pone.0101878

**Published:** 2014-07-18

**Authors:** We-Kang Lee, Yi-An Su, Tzu-Jiun Song, Yao-Chu Chiu, Ching-Hung Lin

**Affiliations:** 1 Department of Psychology, Soochow University, Taipei, Taiwan; 2 Department of Psychology, National Chengchi University, Taipei, Taiwan; 3 Biomedical Engineering R&D Center, China Medical University Hospital, Taichung, Taiwan; 4 Biomedical Electronics Translational Research Center, National Chiao Tung University, Hsinchu, Taiwan; 5 Department of Psychology, Kaohsiung Medical University, Kaohsiung, Taiwan; University of Udine, Italy

## Abstract

The Iowa Gambling Task (IGT) developed by Bechara *et al.* in 1994 is used to diagnose patients with Ventromedial Medial Prefrontal Cortex (VMPFC) lesions, and it has become a landmark in research on decision making. According to Bechara *et al.*, the manipulation of progressive increments of monetary value can normalize the performance of patients with VMPFC lesions; thus, they developed a computerized version of the IGT. However, the empirical results showed that patients' performances did not improve as a result of this manipulation, which suggested that patients with VMPFC lesions performed myopically for future consequences. Using the original version of the IGT, some IGT studies have demonstrated that increments of monetary value significantly influence the performance of normal subjects in the IGT. However, other research has resulted in inconsistent findings. In this study, we used the computerized IGT (1X-IGT) and manipulated the value contrast of progressive increments (i.e., by designing the 10X-IGT, which contained 10 times of progressive increment) to investigate the influence of value contrast on the performance of normal subjects. The resulting empirical observations indicated that the value contrast (1X- vs. 10X-IGT) of the progressive increment had no effect on the performance of normal subjects. This study also provides a discussion of the issue of value in IGT-related studies. Moreover, we found the “prominent deck B phenomenon” in both versions of the IGT, which indicated that the normal subjects were guided mostly by the gain-loss frequency, rather than by the monetary value contrast. In sum, the behavioral performance of normal subjects demonstrated a low correlation with changes in monetary value, even in the 10X-IGT.

## Background

The Iowa Gambling Task (IGT) was developed by Bechara *et al.* [1] to examine the differences in decision making between patients with ventromedial prefrontal cortex (VMPFC) lesions and normal subjects and to prove the somatic marker hypothesis (SMH), according to which people may be guided by their emotions to make fast and rational decisions under complex and uncertain situations [2]. IGT serial studies [1], [3], [4], [5], [6] specifically indicated that because of the effects of brain lesions on emotional processing, patients with VMPFC lesions are unable to form somatic markers (indexed by skin conductance response (SCR)) that can guide people in making rational decisions, and consequently, perform myopically in decision-making tasks [3], [4], [5], [6].

In the original IGT, participants were presented with four decks of cards (i.e., decks A, B, C, and D) and required to choose cards manually. The gain-loss structure of the ten selections in each deck is designed as a section and is repeated four times. The expected value in each section of the original IGT is steady and unchanged. For each selection, participants are informed of the outcome of wins and losses. Although each selection in disadvantageous decks A and B carries an immediate reward of $100, some trials also contain an uncertain punishment (losses of $150 to $1250). Over the long run, selecting from decks A and B causes negative outcomes. In contrast, every selection in advantageous decks C and D carries an immediate reward of $50, but in some trials also contains an uncertain punishment (losses of $50 to $250). Over the long run, selections from decks C and D cause positive outcomes. Furthermore, decks A and C contain 5 losses in every section, but decks B and D have only 1 loss (see Table 1). According to the prediction of the SMH, normal participants will avoid the bad decks and select more from the good decks due to the formation of somatic markers. The results reported by Bechara *et al.* [1], [4] proved the SMH; however, the performance of patients with VMPFC lesions showed a reversed outcome (i.e., they preferred decks A and B over decks C and D).

**Table 1:** The gain-loss structure of original IGT.

	Deck A (Bad)	Deck B (Dad)	Deck C (Good)	Deck D (Good)
Gain in each selection	100	100	50	50
**1**	0	0	0	0
**2**	0	0	0	0
**3**	−150	0	−50	0
**4**	0	0	0	0
**5**	−300	0	-50	0
**6**	0	0	0	0
**7**	−200	0	−50	0
**8**	0	0	0	0
**9**	−250	−1250	−50	0
**10**	−350	0	−50	−250
***Expected value***	**−** ***250***	**−** ***250***	***250***	***250***
***Structure***	***10 gains 5 losses***	***10 gains 1 loss***	***10 gains 5 losses***	***10 gains 1 loss***

Modified from Bechara *et al.* [1]

The IGT researchers demonstrated that the decision-making deficit in patients with VMPFC lesions may be due to insensitivity to future consequences (see details in [5]). Furthermore, in their attempt to normalize the performance of patients with VMPFC lesions by enhancing the patients' sensitivity to future consequences, Bechara *et al.* increased the prospective rewards and penalties gradually in each section of the original IGT and developed a computerized version of the IGT, thereby increasing the expected value in each section of the computerized IGT dynamically. The gains in decks A and B and decks C and D were programmed at an average of $100 or $50, respectively, in the first section of 10 cards and progressively increased by $10 or $5 in consecutive sections in decks A and B and decks C and D, respectively. Additionally, in each section of decks A and C, an additional loss trial was added, but the value remains the same (with losses ranging from $150 to $350 in deck A and $25 to $75 in deck C, which is identical to the original IGT). Therefore, 5 losses are contained in the first section, 6 in the second section, and so on. In each section of decks B and D, the value increases, but the frequency remains the same. The net difference (the expected value of a section) of decks A and B increases by $150 in a negative direction, whereas decks C and D increase by $25 in a positive direction from the second to the sixth section [5], [7] (see Table 2). Although Bechara *et al.* thought that the progressive increment would improve sensitivity to future consequences in patients with VMPFC lesions and normalize their performances, this manipulation did not succeed. Despite the changes made to the original IGT, the empirical results showed that patients with VMPFC lesions continued to perform myopically, preferring the bad decks A and B [5]. This result further showed the decision-making deficit of future-consequence insensitivity resulting from VMPFC lesions.

**Table 2:** The gain-loss structure of 1X-IGT (Bechara *et al.*, 2000).

	Decks	A	B	C	D
**1^st^ section**	**Average gain**	**100**	**100**	**50**	**50**
	1				
	2				
	3	−150		−50	
	4				
	5	−300		−50	
	6				
	7	−200		−50	
	8				
	9	−250	−1250	−50	
	10	−350		−50	−250
	***Expected value*** *	**−** ***250***	**−** ***250***	***250***	***250***
	***Gain-loss frequency***	***10 gains 5 losses***	***10 gains 1 loss***	***10 gains 5 losses***	***10 gains 1 loss***
**2^nd^ section**	**Average gain**	**110**	**110**	**55**	**55**
	11				
	12	−350		−25	
	13			−75	
	14	−250	−1500		
	15	−200		−25	
	16				
	17	−300		−25	
	18	−150		−75	
	19	−250			
	20			−50	−275
	***Expected value***	**−** ***400***	**−** ***400***	***275***	***275***
	***Gain-loss frequency***	***10 gains 6 losses***	***10 gains 1 loss***	***10 gains 6 losses***	***10 gains 1 loss***
**3^rd^ section**	**Average gain**	**120**	**120**	**60**	**60**
	21	−250	−1750		
	22	−300		−25	
	23				
	24	−350		−50	
	25			−25	
	26	−200		−50	
	27	−250			
	28	−150		−25	
	29	−250		−75	−300
	30			−50	
	***Expected value***	**−** ***550***	**−** ***550***	***300***	***300***
	***Gain-loss frequency***	***10 gains 7 losses***	***10 gains 1 loss***	***10 gains 7 losses***	***10 gains 1 loss***
**4^th^ section**	**Average gain**	**130**	**130**	**65**	**65**
	31	−350		−25	
	32	−200	−2000		
	33	−250		−25	
	34	−250		−25	
	35	−150		−25	−325
	36				
	37	−150		−75	
	38	−300		−25	
	39	−350		−50	
	40			−75	
	***Expected value***	**−** ***700***	**−** ***700***	***325***	***325***
	***Gain−loss frequency***	***10 gains 8 losses***	***10 gains 1 loss***	***10 gains 8 losses***	***10 gains 1 loss***
**5^th^ section**	**Average gain**	**140**	**140**	**70**	**70**
	41	−350		−25	
	42	−200			
	43	−250		−25	
	44	−250		−25	
	45	−150		−25	−350
	46		−2250	−25	
	47	−150		−75	
	48	−300		−25	
	49	−350		−50	
	50	−250		−75	
	***Expected value***	**−** ***850***	**−** ***850***	***350***	***350***
	***Gain-loss frequency***	***10 gains 9 losses***	***10 gains 1 loss***	***10 gains 9 losses***	***10 gains 1 loss***
**6^th^ section**	**Average gain**	**150**	**150**	**75**	**75**
	51	−350		−25	
	52	−200		−25	
	53	−250		−25	
	54	−250		−25	
	55	−150		−25	
	56	−250		−25	
	57	−150		−75	
	58	−300	−2500	−25	−375
	59	−350		−50	
	60	−250		−75	
	***Expected value***	**−** ***1000***	**−** ***1000***	***375***	***375***
	***Gain-loss frequency***	***10 gains 10 losses***	***10 gains 1 loss***	***10 gains 10 losses***	***10 gains 1 loss***
	**Final Outcome**	**−3750**	**−3750**	**1875**	**1875**

sadadan both versions s also performed to analyze the effects of gender on each version of IGT.

*Note: Expected value of decks A and B was increased -150 while EV of decks C and D was increased 25 in each section.

1^st^ section: Deck A: −250 + 0*(−150)  =  −250; Deck B: −250 + 0*(−150)  =  −250; Deck C: 250 + 0*(25)  =  250; Deck D: 250 + 0*(25)  =  250

2^nd^ section: Deck A: −250 + 1*(−150)  =  −400; Deck B: −250 + 1*(−150)  =  −400; Deck C: 250 + 1*(25)  =  275; Deck D: 250 + 1*(25)  =  275

3^rd^ section: Deck A: −250 + 2*(−150)  =  −550; Deck B: −250 + 2*(−150)  =  −550; Deck C: 250 + 2*(25)  =  300; Deck D: 250 + 2*(25)  =  300

4^th^ section: Deck A: −250 + 3*(−150)  =  −700; Deck B: −250 + 3*(−150)  =  −700; Deck C: 250 + 3*(25)  =  325; Deck D: 250 + 3*(25)  =  325

5^th^ section: Deck A: −250 + 4*(−150)  =  −850; Deck B: −250 + 4*(−150)  =  −850; Deck C: 250 + 4*(25)  =  350; Deck D: 250 + 4*(25)  =  350

6^th^ section: Deck A: −250 + 5*(−150)  =  −1000; Deck B: −250 + 5*(−150)  =  −1000; Deck C: 250 + 5*(25)  =  375; Deck D: 250 + 5*(25)  =  375

To our knowledge, only a few studies on the influence of value magnitude on selections in IGT exist. Notably, Tomb *et al.* [8] noted two factors, monetary value and long-term outcome, that were confounded in guiding decision making in the IGT. Thus, Tomb *et al.* retested the original findings of the IGT [1], [4] and further changed the schedule in which advantageous decks correlated with large gain-loss values, whereas the bad decks correlated with small gain-loss values. The results of their study showed the value-modification of the gain-loss structure to be critical in driving the anticipatory SCR of the IGT, although the behavioral performance still showed the dominance of the final outcome. On the other hand, by using the original version of the IGT, van den Bos *et al.* [9] suggested that if the ratio of gain value between the good and bad decks increased from 50:100 to 50:200 and 50:300, the selection of bad decks would also increase accordingly (see also [10]). However, Li [11] retested the study by van den Bos *et al.* and showed normal subjects to be insensitive to the changes in gain value. The value-related IGT studies of Tomb *et al.*, van den Bos *et al.*, and Li used the original IGT as the research instrument, which contains stable expected values in each section.

In this study, we used the computerized IGT [5] (1X-IGT), which contains dynamic expected values and further manipulates value contrast. We also designed the 10X-IGT, which contains 10 times the monetary value of progressive changes. Our study revised both gain and loss structures, rather than the gain structure only [9], and it also renders the differences between the net gains in decks C and D and the net losses in decks A and B even larger to test the influence of value contrast on the performance of normal subjects and whether this manipulation facilitates their performances. If the monetary value contrast were a critical factor in guiding choice behavior, the normal subjects' performance should be largely influenced by the huge amount of progressive increments contained in the 10X-IGT rather than the one in the 1X-IGT. Although the EV difference between disadvantageous and advantageous decks are even greater, normal subjects will choose more cards from the advantageous decks (C and D) in the 10X-IGT, compared to the 1X-IGT. Conversely, if the value contrast does not play an important role in guiding decision making, then there is no significant difference between the two versions.

## Materials and Methods

A total of 104 undergraduate students (age: *M*  =  21.42, *SD*  =  3.75) at Soochow University were recruited in this study. Students who participated in this study were given course credit in return. This behavioral study was approved by the Research Ethics Committee of the Psychology Department at Soochow University (REC no: 101-1-2) and was conducted in accordance with the unwritten rule of the Taiwan Psychological Society. All subjects gave written informed consent prior to participating. Moreover, the data were analyzed in group level and reported anonymously. A debriefing was conducted after the experiment was completed. The between-subject design was used here to prevent the practice effect during the game.

### 1X-IGT

52 subjects (26 males) (age: *M*  =  21.39, *SD*  =  3.64) participated in the 1X-IGT experiment. The detailed gain-loss structure of the 1X-IGT was retrieved from the IGT software published by Bechara [7]. In the 1X-IGT, the average gain in decks A and B increases by $10 and the total loss also increases by $250 in each section. Thus, each section in decks A and B has a net loss outcome (i.e., expected value of deck A and B corresponding to Table 2). For example, the net loss in the first section is $250 and in each following section an increment of $150 is added until the net loss reaches $1000 in the sixth section. On the other hand, the average gain in decks C and D has an increment of $5 and the total loss increases by $25 in each section. Therefore, decks C and D have a net gain outcome (i.e., expected value of deck C and D corresponding to Table 2) in each section. For example, the net gain in the first 10 selections is $250, and in the subsequent sections the net gain increases by $25 until it reaches $375 in the sixth section. Furthermore, the losses in decks A and C change in frequency, whereas those in decks B and D change in value (Table 2).

### 10X-IGT

Another 52 subjects (26 males) (age: *M*  =  21.46, *SD*  =  3.88) participated in the 10X-IGT experiment. In this version of the IGT, the average gain in decks A and B increases by $100, and the total loss also increases to $2500 in each section. Therefore, each section of decks A and B has a net loss outcome (i.e., expected value of deck A and B corresponding to Table 3). For example, the net loss in the first section is $250 and increases at an increment of $1500 in each subsequent section until the net loss reaches $7750 in the sixth section. On the other hand, the average gain in decks C and D has a $50 increment, and the total loss increases by $250 in each section. Therefore, decks C and D have a net gain outcome (i.e., expected value of deck C and D corresponding to Table 3) in each section. For example, the net gain in the first ten selections is $250, and in the subsequent section the net gain increases by $250 until it reaches $1500 in the sixth section. Briefly, the difference between the 10X-IGT and the 1X-IGT is the increment of 10 times within the average gain and average loss in each section of every deck. Furthermore, the 10X-manipulation consequently makes the original net gain (decks C and D) or loss (decks A and B) in each section of the 1X-IGT increase by 10 times (see detailed in footnotes in Table 3). Notably, in the process of adjustment, we rounded the gain-loss values into whole numbers but without influencing the gain-loss outcome in each of the sections mentioned above. Two versions of the IGT were administered in semi-groups. The subjects were required to earn money or avoid losing money as much as possible in these two tasks, and the tasks ended after 100 selections were made, which was unknown to the subjects. Both versions of the IGT were programmed using Matlab 7.0 for data collection.

**Table 3:** The gain−loss structure of 10X-IGT.

	Decks	A	B	C	D
**1^st^ section**	**Average gain**	**100**	**100**	**50**	**50**
	1				
	2				
	3	−150		−50	
	4				
	5	−300		−50	
	6				
	7	−200		−50	
	8				
	9	−250	−1250	−50	
	10	−350		−50	−250
	***Expected value*** *	**−** ***250***	**−** ***250***	***250***	***250***
	***Gain-loss frequency***	***10 gains 5 losses***	***10 gains 1 loss***	***10 gains 5 losses***	***10 gains 1 loss***
**2^nd^ section**	**Average gain**	**200**	**200**	**100**	**100**
	11				
	12	−875		−50	
	13			−150	
	14	−625	−3750		
	15	−500		−50	
	16				
	17	−750		−50	
	18	−375		−150	
	19	−625			
	20			−50	−500
	***Expected value***	**−** ***1750***	**−** ***1750***	***500***	***500***
	***Gain-loss frequency***	***10 gains 6 losses***	***10 gains 1 loss***	***10 gains 6 losses***	***10 gains 1 loss***
**3^rd^ section**	**Average gain**	**300**	**300**	**150**	**150**
	21	−895	−6250		
	22	−1070		−70	
	23				
	24	−1250		−140	
	25			−70	
	26	−715		−140	
	27	−895			
	28	−535		−70	
	29	−890		−210	−750
	30			−50	
	***Expected value***	**−** ***3250***	**−** ***3250***	***750***	***750***
	***Gain-loss frequency***	***10 gains 7 losses***	***10 gains 1 loss***	***10 gains 7 losses***	***10 gains 1 loss***
**4^th^ section**	**Average gain**	**400**	**400**	**200**	**200**
	31	−1550		−75	
	32	−920	−8750		
	33	−1130		−75	
	34	−1130		−75	
	35	−710		−75	−1000
	36				
	37	−710		−240	
	38	−1045		−75	
	39	-1555		−150	
	40			−235	
	***Expected value***	**−** ***4750***	**−** ***4750***	***1000***	***1000***
	***Gain−loss frequency***	***10 gains 8 losses***	***10 gains 1 loss***	***10 gains 8 losses***	***10 gains 1 loss***
**5^th^ section**	**Average gain**	**500**	**500**	**250**	**250**
	41	−1750		−90	
	42	−1000			
	43	−1250		−90	
	44	−1250		−90	
	45	−750		−90	−1250
	46		−11250	−90	
	47	−750		−265	
	48	−1500		−90	
	49	−1750		−180	
	50	−1250		−265	
	***Expected value***	**−** ***6250***	**−** ***6250***	***1250***	***1250***
	***Gain-loss frequency***	***10 gains 9 losses***	***10 gains 1 loss***	***10 gains 9 losses***	***10 gains 1 loss***
**6^th^ section**	**Average gain**	**600**	**600**	**300**	**300**
	51	−1925		−100	
	52	−1100		−100	
	53	−1375		−100	
	54	−1375		−100	
	55	−825		−100	
	56	−1375		−100	
	57	−825		−300	
	58	−1650	−13750	−100	−1500
	59	−1925		−200	
	60	−1375		−300	
	***Expected value***	**−** ***7750***	**−** ***7750***	***1500***	***1500***
	***Gain-loss frequency***	***10 gains 10 losses***	***10 gains 1 loss***	***10 gains 10 losses***	***10 gains 1 loss***
	**Final Outcome**	**−24000**	**−24000**	**5250**	**5250**

*Note: Expected value of decks A and B was increased −1500 (i.e. −150*10) while EV of decks C and D was increased 250 in each section; the contrast of final outcome between good and bad decks was larger than that of 1X-version.

1^st^ section: Deck A: −250 + 0*(−150*10)  =  −250; Deck B: −250 + 0*(−150*10)  =  −250; Deck C: 250 + 0*(25*10)  =  250; Deck D: 250 + 0*(25*10)  =  250

2^nd^ section: Deck A: −250 + 1*(−150*10)  =  −1750; Deck B: −250 + 1*(−150*10)  =  −1750; Deck C: 250 + 1*(25*10)  =  500; Deck D: 250 + 1*(25*10)  =  500

3^rd^ section: Deck A: −250 + 2*(−150*10)  =  −3250; Deck B: −250 + 2*(−150*10)  =  −3250; Deck C: 250 + 2*(25*10)  =  750; Deck D: 250 + 2*(25*10)  =  750

4^th^ section: Deck A: −250 + 3*(−150*10)  =  −4750; Deck B: −250 + 3*(−150*10)  =  −4750; Deck C: 250 + 3*(25*10)  =  1000; Deck D: 250 + 3*(25*10)  =  1000

5^th^ section: Deck A: −250 + 4*(−150*10)  =  −6250; Deck B: −250 + 4*(−150*10)  =  −6250; Deck C: 250 + 4*(25*10)  =  1250; Deck D: 250 + 4*(25*10)  =  1250

6^th^ section: Deck A: −250 + 5*(−150*10)  =  −7750; Deck B: −250 + 5*(−150*10)  =  −7750; Deck C: 250 + 5*(25*10)  =  1500; Deck D: 250 + 5*(25*10)  =  1500

### Analysis

The data analysis was conducted using IBM SPSS 20.0. Here, we provided the four-factor ANOVA (version: 1X vs. 10X; sex: male vs. female; decks: ABCD and blocks: 1-5) by using univariate ANOVA analysis to evaluate the main effect of each variable and the interactions among factors. Furthermore, we conducted post-hoc analysis (simple main effects) to understand the influences that contribute to the significant interactions.

## Results

### Global analysis of all factors (version, sex, decks, and blocks)

**Figure 1:**
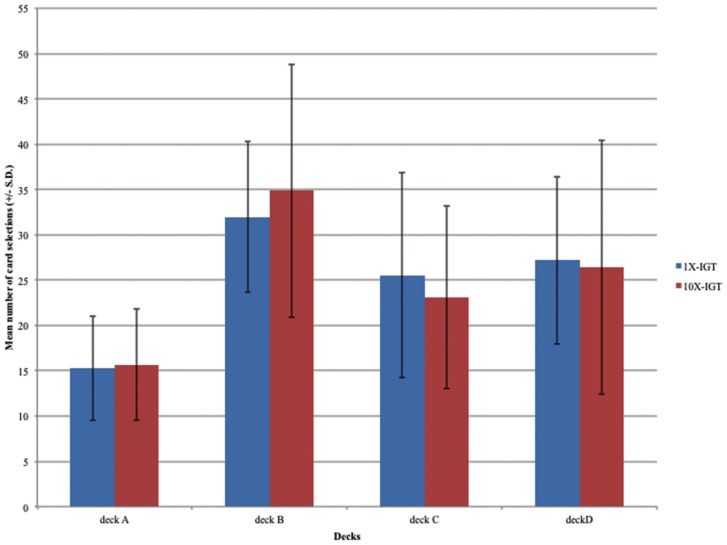
Mean number of deck selection in the two versions of the IGT. Blue bars represent the mean number of cards chosen in the 1X-IGT and red bars represent the mean number of cards chosen in the10X-IGT. In the 1X-IGT, the average number of selections (SD) for decks A, B, C, and D were 15.28 (5.75), 31.98 (8.32), 25.54 (11.32), and 27.20 (9.25), respectively. In the 10X-IGT, the average number of selections were 15.64 (6.15), 34.87 (13.97), 23.12 (10.10), and 26.39 (14.00), respectively. It can be observed that the number of selections from deck B was significantly higher than the averages for the other decks. Normal subjects demonstrated the “prominent deck B phenomenon” in both versions of the IGT. There were no significant differences between the two versions of the IGT. The behavioral performances in the two versions of the IGT were similar, indicating that the manipulation of value contrast in the 10X-IGT did not influence the performance of normal subjects.

**Figure 2:**
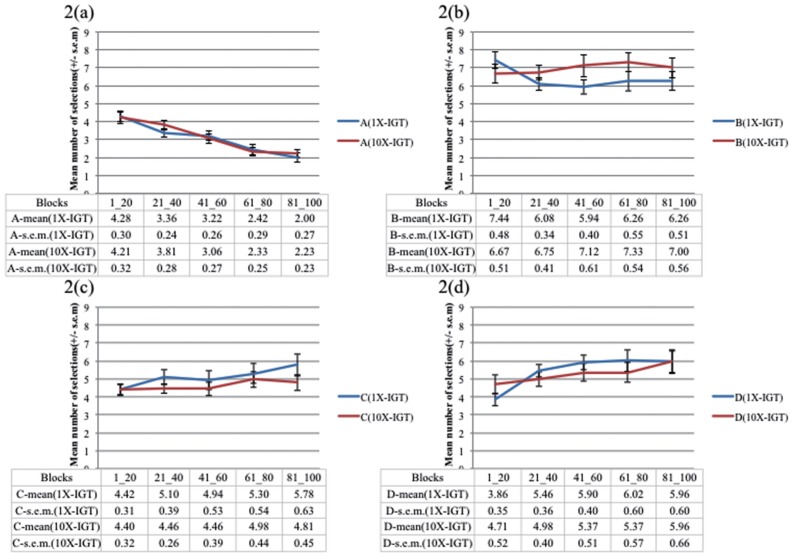
Card selection tendency in 1X-IGT and 10X-IGT. The selection tendencies for (a) deck A, (b) deck B, (c) deck C, and (d) deck D were similar in the 1X-IGT (blue line) and 10X-IGT (red line). The empirical results showed that the manipulation of value contrast in progressive changes was unable to influence the performance of normal subjects. Selection curves in both IGT versions also demonstrated that bad deck B and good decks C and D were preferred by the normal decision makers throughout the game. However, the bad deck A was avoided in most of the blocks (i.e., 20 consecutive selections). Clearly, the selection curve of deck B was unexpectedly higher than that of deck A from the beginning to the end. This phenomenon clearly violates the basic assumption of the IGT.

Two subjects of the 1X-IGT group were excluded from the analysis due to 100 selections of deck C or D. The results of the four factors of the ANOVA (versions [1X vs 10X] × sex [male vs female] × decks [ABCD] × blocks [1]-[5]) indicated no main effects for version, sex, and blocks, but it did for decks (*F* (3, 1960)  =  113.914, *p* < .01) (Fig. 1, 2). Two significant interactions were observed (version * decks: *F* (3, 1960)  =  2.66, *p*  =  .047; decks * blocks: *F* (12, 1960)  =  4.793, p < .01). However, interactions of gender * decks (*F* (3, 1960)  =  1.914, *p*  =  .125), version * gender * decks (*F* (3, 1960)  =  1.320, *p*  =  .266), version * decks * blocks (*F* (12, 1960)  =  1.059, *p*  =  .392), gender * decks * blocks (*F* (12, 1960)  =  1.270, *p*  =  .230) and version * gender * decks * blocks (*F* (12, 1960)  =  .663, *p*  =  .788) are non-significant.

### Post-hoc analysis (simple main effects)

We further conducted post-hoc analysis (simple main effects) by using one way ANOVA to understand the influences that contribute to the significant interactions of version * decks and decks * blocks respectively [12]. The post-hoc analysis showed that, in the interaction of version * decks, there are significant between the decks within the 1X-IGT (*F* (3, 996)  =  50.828, *p* < .01) and 10X-IGT (*F* (3, 1036)  =  64.071, *p* < .01), respectively, but no significant differences between the decks across the two versions (A: *F* (1, 508)  =  .149, *p*  =  .700; B: *F* (1, 508)  =  3.378, *p*  =  .067; C: *F* (1, 508)  =  3.076, *p*  =  .080; D: *F* (1, 508)  =  .251, *p*  =  .616). Detailed paired comparison (corrected by Bonferroni) of decks in each version was listed in Table S1. In both 1X- and 10X-IGT, the preference for deck A was significantly lower than the other decks (*p* < .01), whereas the number of selections for deck B was significantly higher than the selections for decks C (*p* < .01) and D (*p* < .01)(see Fig. 1).

On the other hand, post-hoc analysis of decks * blocks interactions showed that there are significant differences within the blocks in deck A (*F* (4, 505)  =  20.878, *p* < .01) and also within the blocks in deck D (*F* (4, 505)  =  3.270, *p* < .05), but no significant differences within the blocks in decks B (*F* (4, 505)  =  .482, *p*  =  .749) and deck C (*F* (4, 505)  =  1.285, *p*  =  .275). Analysis of decks in fixed block (1–5) also demonstrated difference between decks is significant in each block (block 1: *F* (3, 404)  =  23.337, *p* < .01); block 2: *F* (3, 404)  =  23.622, *p* < .01; block 3: *F* (3, 404)  =  21.836, *p* < .01); block 4: *F* (3, 404)  =  29.884, *p* < .01); block 5: *F* (3, 404)  =  30.971, *p* < .01)).

### 1X- and 10X- IGT learning curve

Notably, the results showed that the interaction of version * decks * blocks (*F* (12, 1960)  =  1.059, *p*  =  .392) was non-significant. The deck by deck comparison between 1X- and 10X-IGT is shown in Figure 2. Based on the traditional IGT analysis [5], we further detailed the deck preference with learning curve format in each version. 1X- IGT learning curve: The analysis of blocks in each deck of the two versions showed that selections from deck A gradually decreased, whereas selections from deck D gradually increased over blocks in the 1X-IGT (A: *F* (4, 196)  =  13.438, *p* < .01; B: *F* (4, 196)  =  1.843, *p*  =  .122; C: *F* (4, 196)  =  1.402, *p*  =  .235; D: *F*(4, 196)  =  4.193, *p* < .01) (Fig. 2).10X-IGT learning curve: Only selections from deck A gradually decreased over decks (A: *F* (4, 204)  =  13.911, *p* < .01; B: *F* (4, 204)  =  .441, *p*  =  .779; C: *F* (4, 204)  =  .809, *p*  =  .521; D: *F*(4, 204)  =  1.270, *p*  =  .283) (Fig. 2).

## Discussion

The empirical results of the four-factor ANOVA indicated no significant differences between the two versions of the IGT (1X vs. 10X). Clearly, the main manipulation was unfounded, and the present observation is inconsistent with the fundamental assumption based on expected value [1], [3], [4], [5], [6]. Also, we found no statistical significance for differences in sex and the block effect. Nevertheless, significant results were observed for decks. Furthermore, the effects of interaction were observed not only in the versions and decks, but also in decks and blocks. Table S1 shows that cards from the bad deck A were chosen less frequently than those from the other three decks in both 1X- and 10X-IGT. Notably, the bad deck B was selected at a significantly higher frequency than the other three decks, which is consistent with the “prominent deck B phenomenon” discussed in some previous studies [13]–[17].

This study used the 1X-IGT (i.e., computerized IGT) [5], [7] and the 10X-IGT to observe the performance of normal subjects. It demonstrated that the manipulation of monetary value contrast did not critically influence the performance of normal decision makers. In this study, we designed 10X-IGT that contains 10 times the monetary increment in progressive changes to test the influence of value contrast on the performance of normal subjects. The empirical results indicate that despite manipulating the increment of value contrast in progressive changes, normal subjects still performed myopically for future outcome and preferred the bad deck B (with frequent gains). Bechara *et al.* [5] found patients with VMPFC lesions to be insensitive to the manipulation of progressive increments in the computerized IGT, and this result further proved that patients with VMPFC lesions are myopic toward future results. Although patients with VMPFC dysfunction were not included in our study, the persistence of the normal subjects' preference for deck B in both tasks showed that the normal subjects were also insensitive to progressive increments even when the value contrast had been increased 10 times.

Unlike past IGT studies [8], [9], [11] related to monetary value which used the original IGT that did not contain dynamic increment as a research instrument, our study used a computerized IGT (1X-IGT) to test the effect of value contrast on decision making. van den Bos *et al.* [9] suggested that widening the ratio of gain value between advantageous and disadvantageous decks (changing 50:100 to 50:200, 50:300) would increase selections from the disadvantageous deck. On the other hand, if the ratio was decreased to 50:50, selections from the disadvantageous deck would also decrease [9]. However, Li [11] suggested that the design of 50:50 proposed by van den Bos *et al.* oversimplified the task and eliminated the conflict between advantageous and disadvantageous decks. Therefore, Li designed the manipulation of 50:75 and reexamined the manipulation (50:100, 50:200) proposed by van den Bos *et al.* The empirical result showed there is indifference between the groups. Li (sample size: 20 subjects in each condition, total 20 males and 40 females) further suggested that the phenomenon that was proposed by van den Bos *et al.* (sample size: 50 vs.100 condition: 9 subjects; 50 vs. 200 condition: 12 subjects; 50 vs. 300 condition: 6 subjects) may be accounted by a small sample size. Recently, Penolazzi *et al.* [10] tested 165 subjects by using the original IGT and the modified IGT, which contains the gain ratio of 300:50 (designed by van den Bos *et al.* [9]; sample size: 40 males, 41 females) to investigate personality differences that might affect decision making. Comparing the selection of each deck between the original IGT and the modified IGT, only a marginal significant effect in deck B (p  =  .055) was noted. These empirical results further supported the explanation proposed by Li [11]. On the other hand, the findings in this study are generally consistent with those of Li [11].

Furthermore, the empirical results in both versions of the IGT (1X- and 10X-IGT) verified previous findings, wherein normal subjects preferred the frequent-gain decks [13], [18]–[21], and reexamined the “prominent deck B phenomenon” (i.e., the normal subjects preferred the disadvantageous deck B which has the feature of frequent gains) as suggested by Lin *et al.* [13] (see also [14]–[17]). Despite the fact that the computerized IGT revised the structure through progressive changes, each cycle in decks B and D still retained 10 gains and 1 loss and changed only in the loss value. This fact shows the “prominent deck B phenomenon” found in the original IGT [13]. Moreover, the results indicated that the manipulation of progressive increment neither improved the performance of patients with VMPFC lesions [5], nor influenced the performance of normal subjects.

Recently, Steingroever *et al.* [22] analyzed data from eight groups of normal subjects and found that normal subjects preferred the decks that resulted in infrequent losses. These results were used to challenge the basic assumptions of the IGT. The results in our study support this argument: despite having revised the 1X-IGT and 10X-IGT with progressive increments, normal subjects still preferred decks with frequent gains, or rather, those with infrequent losses. Therefore, both the previous findings and the present study contradicted the basic assumption of the IGT regarding normal subjects.

Based on our empirical results, although monetary value contrast was increased and made the net losses in decks A and B even larger in the negative direction and the net gains in decks C and D even larger in positive direction, no differences in behavioral performance were found in the two versions of the IGT. This suggests that performance in the IGT is dominated by a gain-loss frequency, rather than by value and long-term outcomes [13]–[16], [23]–[27].

Recently, researchers have also illustrated that the primary factor in the performance of normal subjects in the IGT was gain-loss frequency and that expected value was secondary. Moreover, the study also suggested that the results were consistent with those of another study using animal subjects, which showed that the influence of reward frequency was larger than that of reward magnitude on the behavior of animal subjects [27], a statement that is confirmed by our study.

In this study, we examined the influence of sex on performance in both versions of the IGT. The results of our study are inconsistent with those of previous studies which suggested that women preferred decks yielding frequent gains [28]–[31]. Furthermore, in contrast to the original IGT [1] that contains 40 cards in each deck, the computerized IGT [5] contains 60 cards in each deck. Therefore, if the subjects wanted to choose more than 40 cards from each deck of the original IGT (or more than 60 cards from each deck of the computerized IGT), they would be prevented from doing so. However, unlike this setting, the structures of both IGT used in our study were repeated after 60 selections of each deck. This limitation should be noted in future researches.

## Conclusion

This research shows that even normal decision-makers were mostly uninfluenced by the manipulation of value contrast under uncertainty. This observation is partially inconsistent with the finding of van den Bos *et al.* [9], but consistent with those produced in several animal studies and behavioral-analysis literatures. Our study showed the manipulation of progressive increment suggested by Bechara *et al.* [5] to be not only invalid for improving the performance of patients with VMPFC lesions but also for determining the behavior of normal subjects. Moreover, even when the monetary value contrast of the gain-loss structure increased by 10 times, the subjects continued to prefer the decks with frequent gains. Furthermore, the “prominent deck B phenomenon” was demonstrated in both versions of the IGT.

## Supporting Information

Table S1 **Paired comparison (corrected by Bonferroni) for two decks in each version (1X- and 10X-IGT).**
(doc)
