# Supplementary material for: Are Normal Decision-Makers Sensitive to Changes in Value Contrast under Uncertainty? Evidence from the Iowa Gambling Task
Source: PLoS One. 2014 Jul 18;9(7):e101878. doi: 10.1371/journal.pone.0101878 (PMC4103768; doi:10.1371/journal.pone.0101878)
Supplement: Table S1 — Paired comparison (corrected by Bonferroni) for two decks in each version (1X- and 10X-IGT). (doc) [file pone.0101878.s001.doc]

**Table S1** Paired comparison (corrected by Bonferroni) for two decks in each version (1X- and 10X-IGT)

| **1X-IGT** | | |  | **10X-IGT** | | |
| --- | --- | --- | --- | --- | --- | --- |
| **Decks** | **Mean difference** | ***p*-value** |  | **Decks** | **Mean difference** | ***p*-value** |
| **A-B** | -3.340 | .000** |  | **A-B** | -3.846 | .000** |
| **A-C** | -2.052 | .000** |  | **A-C** | -1.496 | .000** |
| **A-D** | -2.384 | .000** |  | **A-D** | -2.150 | .000** |
| **B-C** | 1.288 | .000** |  | **B-C** | 2.350 | .000** |
| **B-D** | .956 | .004** |  | **B-D** | 1.696 | .000** |
| **C-D** | -.332 | 1.000 |  | **C-D** | -.654 | .123 |

Note: *The present comparison was focusing on each two decks of each version.*
***p < .01*
**p < .05*
